# Introduction of Graphene/h-BN Metamaterial as Neutron Radiation Shielding by Implementing Monte Carlo Simulation

**DOI:** 10.3390/ma15196667

**Published:** 2022-09-26

**Authors:** Marzieh Hassanpour, Mehdi Hassanpour, Simin Faghihi, Saeedeh Khezripour, Mohammadreza Rezaie, Parvin Dehghanipour, Mohammad Rashed Iqbal Faruque, Mayeen Uddin Khandaker

**Affiliations:** 1 Space Science Centre (ANGKASA), Institute of Climate Change (IPI), Universiti Kebangsaan Malaysia, Bangi 43600, Malaysia; 2 Department of Engineering, Khorasgan (Isfahan) Branch, Islamic Azad University, Arghavanieh, Isfahan 8155139998, Iran; 3 Department of Molecular and Atomic Physics, Faculty of Modern Science and Technology, Graduate University of Advanced Technology, Kerman 7631885356, Iran; 4 Department of Nuclear Engineering, Faculty of Modern Sciences and Technologies, Graduate University of Advanced Technology, Kerman 7631885356, Iran; 5 Department of Physics, Payame Noor University (PNU), Tehran 1599959515, Iran; 6 Centre for Applied Physics and Radiation Technologies, School of Engineering and Technology, Sunway University, Bandar Sunway, Petaling Jaya 47500, Malaysia; 7 Department of General Educational Development, Faculty of Science and Information Technology, Daffodil International University, DIU Road, Dhaka 1341, Bangladesh

**Keywords:** metamaterial, neutron radiation shielding, current transmission rate, MCNPX, concrete

## Abstract

In this paper, graphene/h-BN metamaterial was investigated as a new neutron radiation shielding (NRS) material by Monte Carlo N-Particle X version (MCNPX) Transport Code. The graphene/h-BN metamaterial are capable of both thermal and fast neutron moderator and neutron absorber process. The constituent phases in graphene/h-BN metamaterial are chosen to be hexagonal boron nitride (h-BN) and graphene. The introduced target was irradiated by an Am–Be neutron source with an energy spectrum of 100 keV to 15 MeV in a Monte Carlo simulation input file. The resulting current transmission rate (CTR) was investigated by the MCNPX code. Due to concrete’s widespread use as a radiation shielding material, the results of this design were also compared with concrete targets. The results show a significant increase in NRS compared to concrete. Therefore, metamaterial with constituent phase’s graphene/h-BN can be a suitable alternative to concrete for NRS.

## 1. Introduction

In recent years, the radiation shielding concept has been extended to exploit radiation-related technology properties, such as radiography and radiotherapy [1]. Neutrons are the most important rays that need further investigation to shield them because these rays do not have an electric charge, and materials that can cause significant growth of cross-sections for neutron radiation shielding (NRS) should be used to moderate and absorb them. One particular class of concrete, concrete containing additives, has attracted much interest because it exhibits a higher radiation shielding as compared to conventional concrete [2,3]. These additives contain green additives such as pistachios shell and date palm leaf [4], boron-based additives such as basalt-boron fiber [5], polyethylene [3], carbon powder [6], led-based glass systems [2], etc. The mechanical properties of concretes containing additives often decline due to the disruption of the concrete-mixing design standard [3,7,8,9]. Therefore, the need to study advanced technologies in this regard can be important. Over the past few years, the application of metamaterials has expanded due to the extraordinary efficiency of unusual mechanical properties, such as ultra-lightweight and stiffness [10,11], negative mass density [12,13], and high-energy absorption [14,15]. As the name implies, metamaterials are synthetic composite materials that exhibit properties that are not inherent to natural materials. In metamaterials, mechanical properties are provided by different construction phases. Co-continuous two-phase composites contain the complete interpenetration of the constituent phases in all three dimensions, each contributing to the composite’s overall properties in a totally independent manner. Nevertheless, all three dimensions of these two phases are intertwined topologically and acted upon mutually [15,16]. Consequently, in comparison to their rod-connected counterparts, triply periodic minimal surface structures with simple cubic lattice exhibit significantly improved elastic properties [17]. Therefore, by designing these metamaterials structures with the proper combination of ingredients phases can help guide the creation of NRS.

Preparation of high-quality metamaterials can be accomplished through laser chemical vapour deposition. Because of its low porosity, high crystallinity, and high purity, the material deposited by this technique usually has mechanical properties and a thermal stability that are superior to other techniques [18]. That is why there have been numerous materials (including carbon and boron) deposited by laser chemical vapour deposition techniques [18]. The atomic thickness of this carbon material provides excellent mechanical and impermeability properties, which make it very useful in many applications [19,20]. Recently, the combination of hexagonal boron nitride (h-BN) and graphene has been known as a metamaterial, and researchers are investigating the properties and characteristics of this metamaterial in applied fields [21,22,23,24,25,26,27].

In this article, the shielding properties of this particular type of metamaterial (graphene/h-BN) have been investigated by Monte Carlo N-Particle Transport Code (MCNPX) and the results compared with concrete as an indicator neutron shield. One of the index codes for evaluating the effects of radiation is the MCNPX code [21,22,23,28,29]. Therefore, the NRS calculations were performed using MCNPX code in this study.

The main aim of this research is the initial step to evaluate metamaterial as a neutron radiation shielding, and for this purpose, the results are compared with the most important neutron radiation shield, concrete, and if it performs better than concrete, it can be introduced as a neutron radiation shielding. It can provide a platform for more calculations and research in line with the use of metamaterials in the radiation shielding industry. By simulating a model, theorists can examine its properties and compare their predictions with the simulation results. The simulation is actually a virtual experiment that is performed to understand the response of the behavior system before a real one is conducted. However, it allows experimentalists to assess the validity of experimental results when a theory is highly complicated, while they do not have a precise method to solve it by theorizing. Since Monte Carlo simulations and modeling can be used in the science of particle transport at no cost, the calculations were performed through coding. As one of the most significant nuclear codes, the MCNPX code has been validated and verified by many researchers [30,31,32], and the results show a high compatibility between simulations and laboratory calculations [33,34,35,36,37,38]. The programming language of the MCNPX code is FORTRAN, but to extract the data, an input file must be written in text format with special commands. As a main part of the MCNPX code input file structure, three types of cards are used: cell cards, surface cards, and data cards.

A blank line is required to separate these three main cards. A cell card is required to describe the geometry. Each cell is made of different surfaces that are listed on the surface card. The data card contains all the necessary information for making the cells, including the material specifications (density and percentage of the elements in it), the specifications of the radiation source and how to extract the data. After writing the MCNPX code input file, this file is executed, and the desired results are recorded in an output file.

## 2. Materials and Methods

By defining the input file (cell card, surface card, data card), the geometric structure of the target was introduced to the MCNPX code. This structure is a 30 × 30 × 30 cm^3^, which is initially completely filled with concrete and then defined with a metamaterial structure. There are two phases in the metamaterial state: hexagonal boron nitride and graphene. Hexagonal boron nitride (h-BN) and graphene both have similar hexagonal layer structural features, so that h-BN is referred to as white graphene.

**Figure 1:**
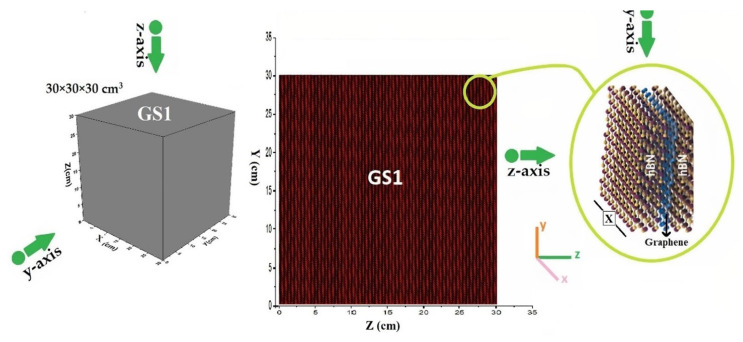
A geometric structure of metamaterial with periodic Macrobody surfaces structures with cubic lattice (GS1) obtained by MCNPX code.

The design of the metamaterial structure was conducted in two ways. Geometric structure 1 (GS1): The 30 × 30 × 30 cm^3^ cube is characterized by triply periodic Macrobody surface structures exhibiting a simple cubic lattice (Figure 1). Macrobody is a term that defines all the surfaces of a certain volume such as sphere, cylinder, and cube inside the MCNPX code [39].

**Figure 2:**
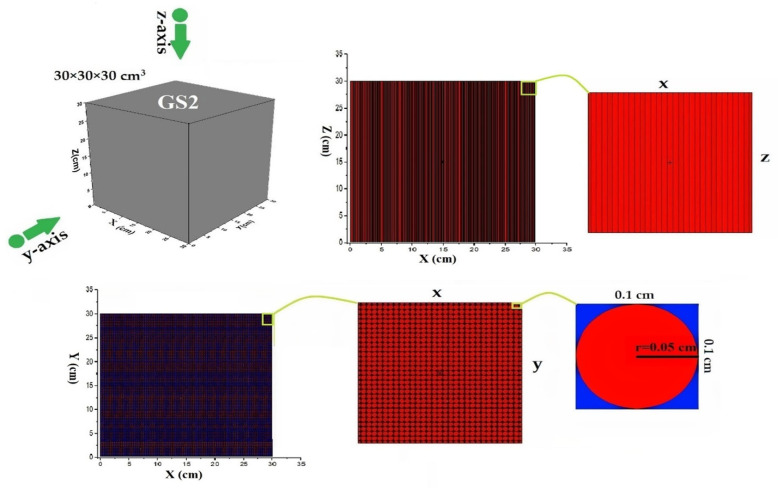
A geometric structure of metamaterial with cubic lattice filled with rod columns (GS2) obtained by MCNPX code.

Geometric structure 2 (GS2): The 30 × 30 × 30 cm^3^ cube is characterized by structures of a periodic cubic lattice, each of them filled with rod columns (Figure 2, Red color) and metamaterial placed in them. These cylindrical rods are placed next to each other, and the surrounding space is filled with air (Figure 2, blue color). The cylindrical rods mean that the metamaterial sheets of the GS1 structure are rolled and form a cylindrical rod structure. As shown in Figure 2, a cylinder with a radius of 0.05 cm was defined inside each cubic lattice structure with dimension of 0.1 × 0.1 × 30 cm^3^ and filled with graphene/h-BN metamaterial.

The “Fill” and “Universe” orders can be used in conjunction with the “Lat” command to specify a lattice type (cubic or hexagonal) in the MCNPX cell cards. The required surfaces are defined on the surface card using the commands including Macrobody Rectangular Parallelepiped (RPP), Macrobody Right Hexagonal Prism (HEX or RHP), Macrobody Right Circular Cylinder (RCC), and sphere with centered at origin (So). A Macrobody has a specific definition, which is called from the code using the above symbols, allowing it to identify all types of Macrobodies [39].

As part of the MCNPX code input file, materials are defined using the ZAID statement, which multiplies an atomic number by a 1000 and plus mass number (Z × 1000 + A) [24]. It should be noted that to define a material that has a combination of different elements, the atomic percentage or weight percentage of each element must be included in the data card. Each of the above geometric structures is filled with materials that are suitable for NRS. For the control sample, the cube defined in the cell card was filled with concrete constituents whose specifications are in accordance with Table 1.

**Figure 3:**
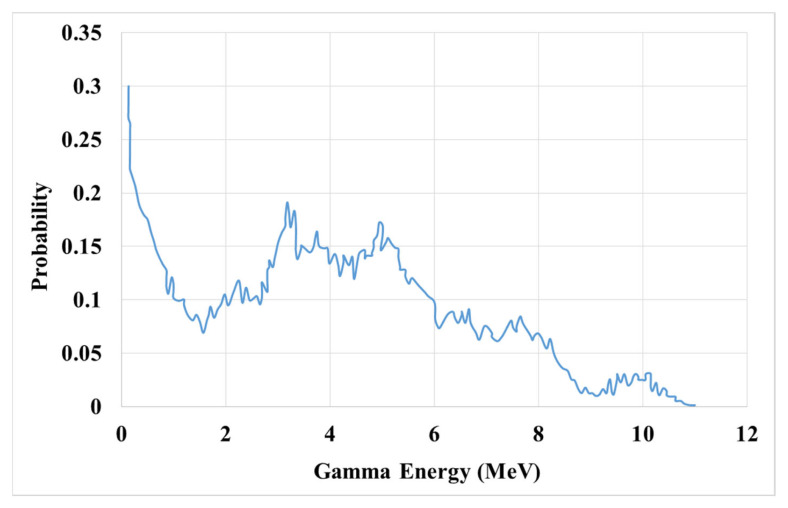
Am–Be neutron source energy spectrum [4].

The constituent phases in each metamaterial simulation are chosen to be graphene hexagonal boron nitride (h-BN) and graphene with their ZAID defined in the data card. The neutron source used in the simulation is Americium–Beryllium (Am–Be), which has an energy range from 100 keV to 15 MeV. In Figure 3, the spectrum of this neutron source is depicted along the energy axis.

To obtain NRC in the MCNPX code, the current transmission rate (CTR) was calculated using F1 tally (surface current tally) and flux mesh tally. The mesh tally was defined in three dimensions and meshes were considered along the direction of the *z*-axis and *y*-axis and aligned with the direction of the incident beam. To consider all scenarios of hitting the target, the direction of the incident beam was considered along the z- and y-axes, since the target structure is different perpendicularly and parallelly to the sheets. For example, if the beam is irradiated in the direction of the *z*-axis in the GS1 structure, the incident beam will be perpendicular to the parallel sheets (xz surface), and if it is irradiated in the direction of the *y*-axis, it will hit the target parallel to the xy sheet. Therefore, both radiation directions were investigated in terms of NRS. Finally, the shielding percentage (SP%) is computed by varying the axis between 0 and 30 cm (cm) and calculating the CTR from an external surface.

## 3. Results and Discussion

**Figure 4:**
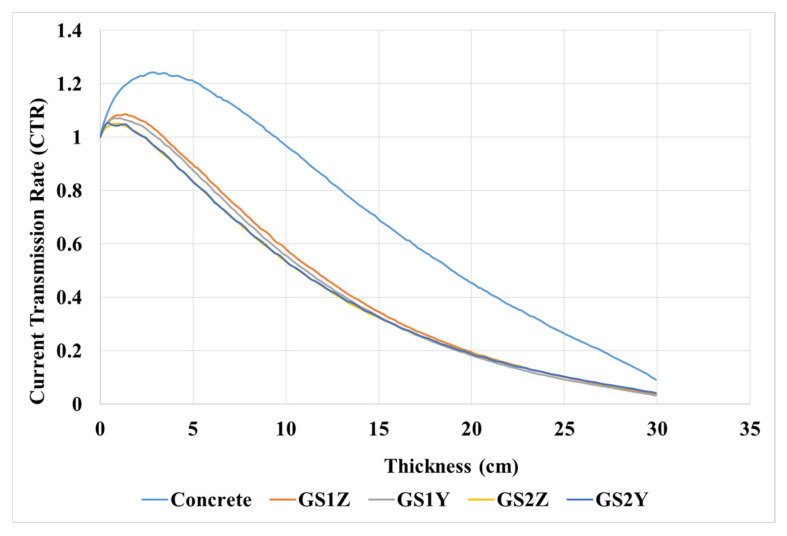
Comparison of CTR obtained from ordinary concrete and GS1.

First, the CTR was evaluated for a cube target with 30 × 30 × 30 cm^3^ sides containing concrete and metamaterial with the GS1 and the GS2 structure. The incident beam was irradiated towards the target in two directions of z- and y-axis, the results of which are reported in Figure 4. GS1Z, GS1Y, GS2Z, and GS2Y, respectively, are the CTR obtained from GS1 per incident beam along the z-axis, the CTR obtained from GS1 per incident beam along the y-axis, the CTR obtained from GS2 per incident beam along the z-axis, and the CTR obtained from GS2 per incident beam along the y-axis.

According to Figure 4, the CTR decreases with increasing the thickness of the targets.

Significant effects of NRS per metamaterial can be observed compared to conventional concrete. As shown in Table 2, at a thickness of 20 cm, the shielding percentage (SP%) of metamaterials is between 80.69% and 81.82%, which is 25.92% higher than the SP% of concrete in the lowest case. By increasing the thickness of the concrete target to 30 cm, the maximum SP% of the concrete is 90.86%, which is extremely close to the SP% of metamaterial at 25 cm thick. However, the metamaterial SP% at a thickness of 30 cm reaches a significant amount of 96.43% in GS1Z and 96.77% in GS1Y.

As the incident beam can enter the target from two different radiation directions, the neutron leakage, which is the CTR, may be affected. This was checked using the μ variable as a percentage difference in the neutron leakage rate, which is defined as follows:

$$\mu =\frac{\mathrm{Neutron}\;\mathrm{leakage}\;\mathrm{in}\;\mathrm{the}\;\mathrm{direction}\;\mathrm{of}\;\mathrm{the}\;z\;\mathrm{axis}-\;\mathrm{neutron}\;\mathrm{leakage}\;\mathrm{in}\;\mathrm{the}\;\mathrm{direction}\;\mathrm{of}\;\mathrm{the}\;y\;\mathrm{axis}}{\mathrm{Neutron}\;\mathrm{leakage}\;\mathrm{in}\;\mathrm{the}\;\mathrm{direction}\;\mathrm{of}\;\mathrm{the}\;z\;\mathrm{axis}}\;\times \;100$$

**Figure 5:**
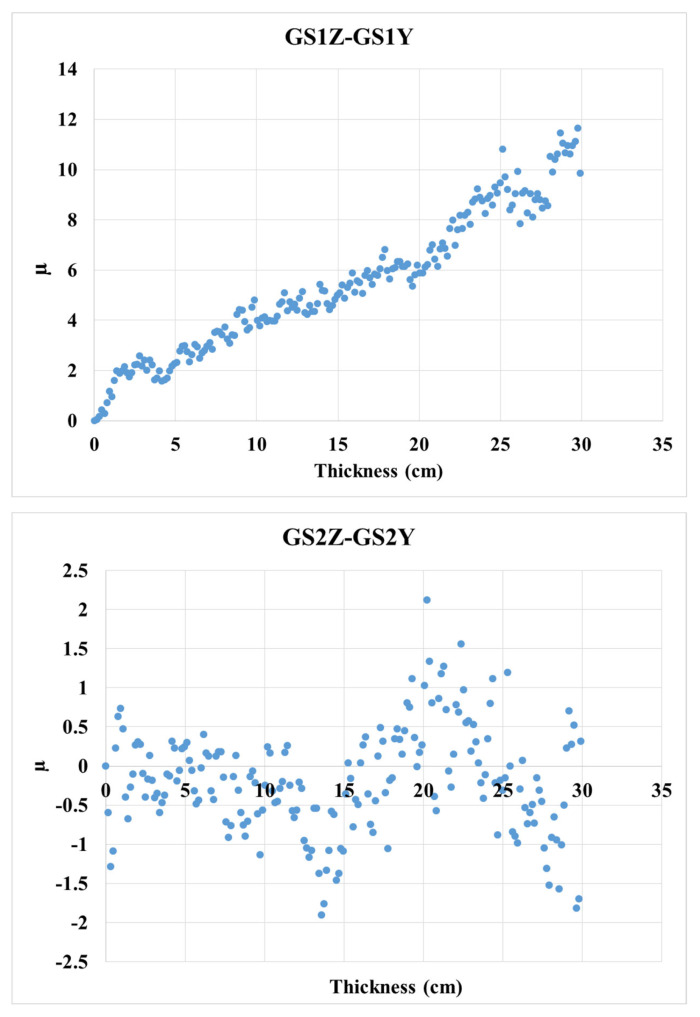
Comparison of CTR obtained from GS1Z−GS1Y and GS2Z−GS2Y.

The results of Figure 5 demonstrate that changing the direction of the radiation of the neutron beam to the layers in both vertical and parallel directions has negligible effects, and this can be explained by the neutron diffusion process that occurs after the initial collision with the target, which causes neutrons to collide at different angles throughout the entire target’s volume.

**Figure 6:**
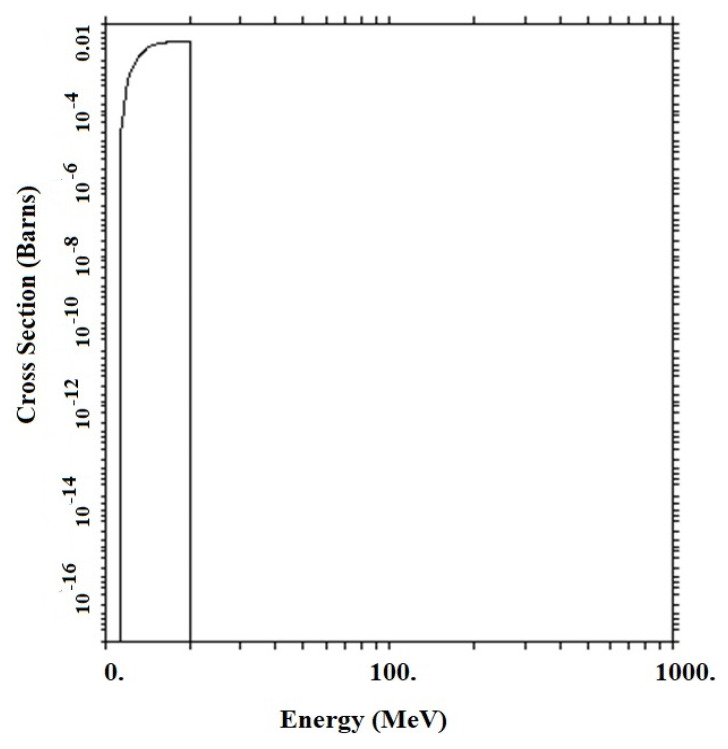
^A^X (n, 2n) ^A^−^1^X cross-section in GS1 and GS2.

**Figure 7:**
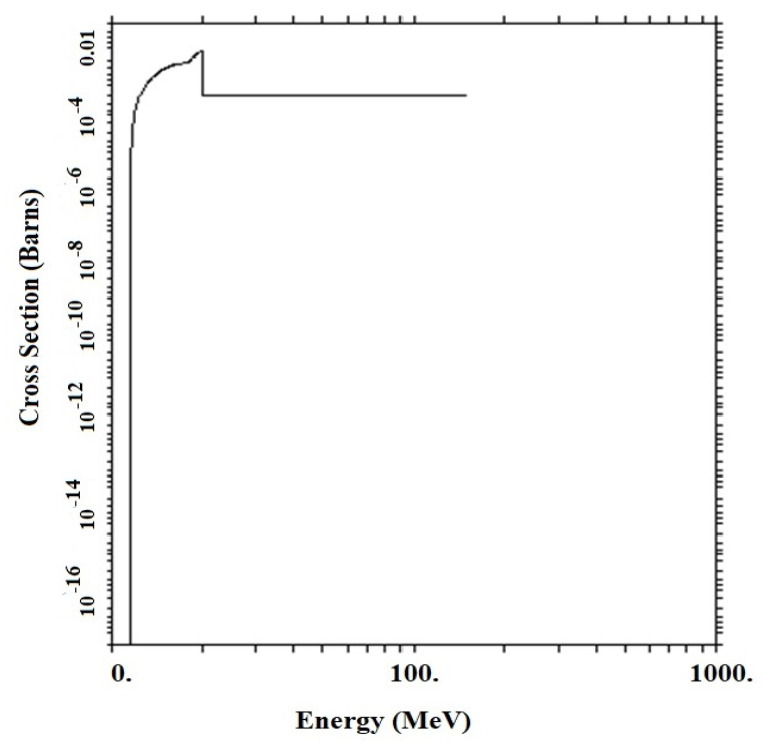
^A^X (n, 2n) ^A^−^1^X cross-section in concrete composite structure.

Accordingly, the optimal geometric structure for the metamaterial with the two phases containing graphene/h-BN was investigated by examining GS1 and GS2. Concrete, the GS1 and GS2 structures in the initial thicknesses have an exponential growth of the CTR, which is due to the production of neutrons per (n, 2n) reaction (Figure 6 and Figure 7). ^A^X (n, 2n) ^A−1^X reactions are performed at GS1 in thicknesses between zero and 3.4 cm, while neutrons are produced at GS2 in thicknesses up to 2.3 cm.

As shown in Table 2, the NRS trend of metamaterial is higher for the GS1 than for the GS2 structure in 30 cm thicknesses, but the SP% ratio (GS1 SP%/GS2 SP%) is 0.99 in the thickness range from 20 to 30 cm. In spite of this, both structures can be suggested for NRS based on their close CTR percentages.

**Figure 8:**
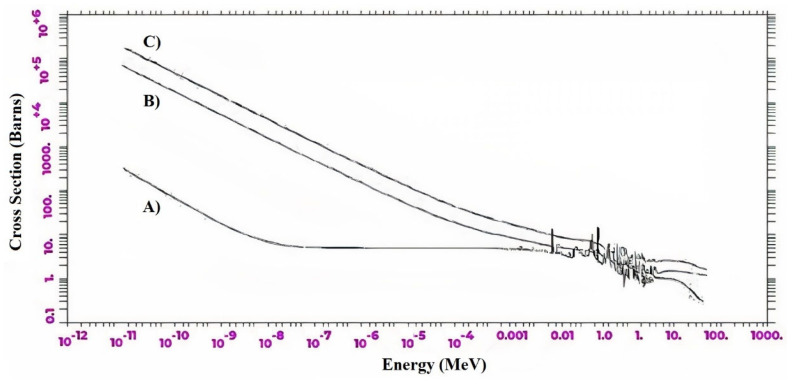
Total neutron cross-section for A) concrete, and metamaterial B) GS2, and C) GS1.

To evaluate the reason for the improvement of NRS in GS1 and GS2 compared to concrete, the cross-section of each of them was calculated and charted in the energy ranges between 10^−12^ and 1000 MeV. According to the results shown in Figure 8, the absorption cross-section of neutrons with energy up to 10^−8^ in GS1 and GS2 reach a climax (almost 100 times), which is a very impressive result and can be attributed to the boron element, which has a highly remarkable neutron absorption cross-section. Furthermore, the cross-sectional area of collision in energy ranges beyond 10^−8^ also has a significant growth. Therefore, the neutrons in the GS1 and GS2 structures first moderate the fast neutrons with a more acceptable scattering cross-section than concrete and then absorb thermal neutrons.

In 2020, Ying et al. [40], investigated the shielding properties of polyethylene/hexagonal boron nitride (polyethylene/h-BN). In their research, a multilayer structure including high-density polyethylene/hexagonal boron nitride layers and low-density polyethylene layers has been introduced. For comparison, this material was also simulated in a cubic structure with dimensions of 30 × 30 × 30 cm^3^.

**Figure 9:**
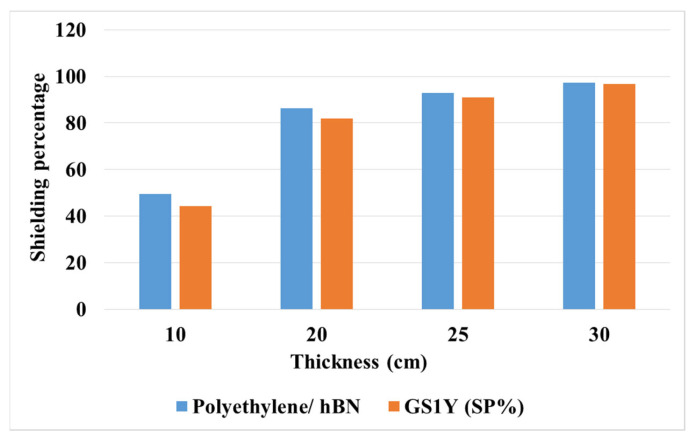
Shielding percentage obtained from polyethylene/h-BN and GS1Y.

While polyethylene/h-BN shows slightly better results, the SP% is almost close to each other in both structures (Figure 9).

One of the most important features of neutron shielding is thermal conductivity (TH). Shielding structures with more TH will therefore be considered advantageous. The value of TH for graphene/h-BN is in the range between 200 and 1000 W/mk [41], while the TH obtained from polyethylene/h-BN is 3.5 at best [40]. Although SP% is slightly better than graphene/h-BN at thicknesses between 5 and 25 cm, graphene/h-BN can be a more valuable and suitable radiation shielding material due to its thermal conductivity range from 57 to 258 times. Nevertheless, it is much more suitable for protecting against neutrons than concrete.

Obtaining a material that performs well as a neutron shield and has high thermal conductivity is the most important application of shielding in cosmic rays. It may therefore be beneficial to introduce graphene/h-BN metamaterial into this research as a platform for wider studies.

## 4. Conclusions

This paper introduces a new neutron radiation shield (NRS) using metamaterial with constituent phase’s hexagonal boron nitride (h-BN) and graphene. To evaluate this structure, the Am–Be neutron source was defined in the input file of the MCNPX code, and the current transmission rate (CTR) of the incident neutrons that enter the target was evaluated. The results of metamaterial were compared with the most famous neutron shield, concrete and polyethylene/hexagonal boron nitride. A maximum NRS of 96.43% and 96.77% was obtained at a thickness of 30 cm for the GS1Z and GS1Y, whereas the concrete led to a maximum NRS of 90.86% at a thickness of 30 cm, which is close to the shielding percentage (SP%) obtained from metamaterial at 20 cm thickness. On the other hand, graphene/h-BN has an extremely high thermal conductivity, making it a very significant advantage for NRS. Therefore, graphene/h-BN metamaterials can be a suitable combination to moderate the speed of fast neutrons and ultimately their absorption.

## Figures and Tables

**Table 1:** Weight percentage of the concrete constituents [4].

Elements	ZAID	Wt% ^1^	Elements	ZAID	Wt% ^1^
H	1001	1.065	Fe	26,056	0.283
O	8016	53.489	Mg	12,024	0.244
Si	14,028	30.116	S	16,032	0.178
Ca	20,040	12.266	Na	11,023	0.07
Al	13,027	0.364	K	19,039	0.078
C	6012	1.847			

^1^ Wt%: weighting percentages (Normalization to Sum 1).

**Table 2:** Shielding percentage obtained from metamaterials and concrete.

Z	Concrete (SP%)	GS1Z (SP%)	GS1Y (SP%)	GS2Z (SP%)	GS2Y (SP%)
10 cm	3.31	42.02	44.34	46.7	46.57
20 cm	54.76	80.69	81.82	81.12	81.31
25 cm	74.08	89.91	91.01	89.92	89.9
30 cm	90.85	96.43	96.77	95.86	95.87

## Data Availability

Not applicable.
